# FSCC: Few-Shot Learning for Macromolecule Classification Based on Contrastive Learning and Distribution Calibration in Cryo-Electron Tomography

**DOI:** 10.3389/fmolb.2022.931949

**Published:** 2022-07-05

**Authors:** Shan Gao, Xiangrui Zeng, Min Xu, Fa Zhang

**Affiliations:** ^1^ High Performance Computer Research Center, Institute of Computing Technology, Chinese Academy of Sciences, Beijing, China; ^2^ University of Chinese Academy of Sciences, Beijing, China; ^3^ Computational Biology Department, School of Computer Science, Carnegie Mellon University, Pittsburgh, PA, United States

**Keywords:** few-shot learning, cryo-ET, macromolecule classification, contrastive learning, distribution calibration

## Abstract

Cryo-electron tomography (Cryo-ET) is an emerging technology for three-dimensional (3D) visualization of macromolecular structures in the near-native state. To recover structures of macromolecules, millions of diverse macromolecules captured in tomograms should be accurately classified into structurally homogeneous subsets. Although existing supervised deep learning–based methods have improved classification accuracy, such trained models have limited ability to classify novel macromolecules that are unseen in the training stage. To adapt the trained model to the macromolecule classification of a novel class, massive labeled macromolecules of the novel class are needed. However, data labeling is very time-consuming and labor-intensive. In this work, we propose a novel few-shot learning method for the classification of novel macromolecules (named FSCC). A two-stage training strategy is designed in FSCC to enhance the generalization ability of the model to novel macromolecules. First, FSCC uses contrastive learning to pre-train the model on a sufficient number of labeled macromolecules. Second, FSCC uses distribution calibration to re-train the classifier, enabling the model to classify macromolecules of novel classes (unseen class in the pre-training). Distribution calibration transfers learned knowledge in the pre-training stage to novel macromolecules with limited labeled macromolecules of novel class. Experiments were performed on both synthetic and real datasets. On the synthetic datasets, compared with the state-of-the-art (SOTA) method based on supervised deep learning, FSCC achieves competitive performance. To achieve such performance, FSCC only needs five labeled macromolecules per novel class. However, the SOTA method needs 1100 ∼ 1500 labeled macromolecules per novel class. On the real datasets, FSCC improves the accuracy by 5% ∼ 16% when compared to the baseline model. These demonstrate good generalization ability of contrastive learning and calibration distribution to classify novel macromolecules with very few labeled macromolecules.

## 1 Introduction

Biological processes in cells are dominated by complex networks of molecular assemblies and their interactions. Analyzing the native structure and spatial distribution of molecular assemblies is essential for revealing the macromolecular mechanism of cellular processes. Cryo-electron tomography (cryo-ET) is an emerging technique for three-dimensional (3D) visualization of macromolecular structures (Lučić et al., 2013; Wan and Briggs, 2016). Compared to other 3D visualization methods such as X-ray (Blanchet and Svergun, 2013), cryo-ET has the advantage of revealing the structure of macromolecular structures in a near-native state at the sub-molecular resolution. Many important native macromolecular structures have been discovered using cryo-ET, such as SARS-Cov-2 that caused the COVID-19 pandemic (Liu et al., 2020).

In principle, the cellular tomograms imaged by cryo-ET can capture millions of macromolecules with diverse structures. To recover structures of macromolecules, diverse macromolecules in tomograms should be detected (Melia and Bharat, 2018), classified (Gao et al., 2021), aligned and averaged (Zeng et al., 2021), and called subtomogram averaging (STA) (Bharat and Scheres, 2016). Macromolecule classification aims to classify diverse macromolecules into structurally homogeneous subsets accurately. The input to macromolecule classification is a subtomogram, a subvolume of the tomogram. Each subtomogram contains a complete macromolecule. The accuracy of macromolecule classification directly affects the performance of downstream tasks. Because misclassified macromolecules can introduce wrong structures, it further increases the difficulty in alignment. However, macromolecule classification remains challenging due to the low signal-to-noise ratio (SNR), a large variety of macromolecular structures, and small size of macromolecules.

One pioneering method for macromolecule classification is the template search (Yu and Frangakis, 2011). Given the template structure, this method calculates the cross-correlation coefficient between each subvolume of the tomogram and the template structure through a sliding window. When the cross-correlation coefficient is higher than a threshold, the target macromolecule is been identified. Though this method has been successfully applied to identify some large macromolecules (Böhm et al., 2000), the performance highly depends on the template structure. When the targets and template structures are from different organisms or have different conformations, these targets can be missed (Moebel et al., 2021). To avoid relying on template structures, template-free classification methods have been developed (Jonić, 2016; Xu et al., 2019; Martinez-Sanchez et al., 2020). For example, Xu et al. (Xu et al., 2019) proposed an iterative clustering process to group macromolecules that have the same macromolecular structures. Although this template-free method can classify novel macromolecules, iterative clustering in 3D is time-consuming. This makes the method only suitable for small datasets and has limited application in practical scenarios.

Recently, the SHREC contest (Gubins et al., 2020) caused a surge in supervised deep learning–based subtomogram classification methods (Himes and Zhang, 2018; Harastani and Jonic, 2021; Moebel et al., 2021; Pyle and Zanetti, 2021). For example, Xu et al. proposed the DoG-3D-CNN (Gubins et al., 2019) method to classify subtomograms after filtering image noise with a difference of Gaussian (DoG) filter (Wang et al., 2012). Considering that the subtomogram is a 3D image, to extract more features from depth dimension, Gao et al. proposed 3D-dilated-Densenet (Gao et al., 2020). 3D-dilated-Densenet improves the classification performance of macromolecules of small size. Despite improving classification accuracy and decreasing the processing time, the abovementioned supervised deep learning–based methods often have one major bottleneck: trained models have limited ability to classify novel macromolecules that are unseen in the training stage. To adapt the trained model to macromolecule classification of a novel class (unseen class in the training stage), massive labeled macromolecules of the novel class are required to retrain the model. This is inefficient and undesirable in practice tasks as labeling macromolecules is time-consuming and laborious (Oda and Kikkawa, 2013). Furthermore, due to complicated structures and distortion caused by missing wedge and noise, it is hard to label macromolecules with naked eyes even by experts.

**FIGURE 1:**
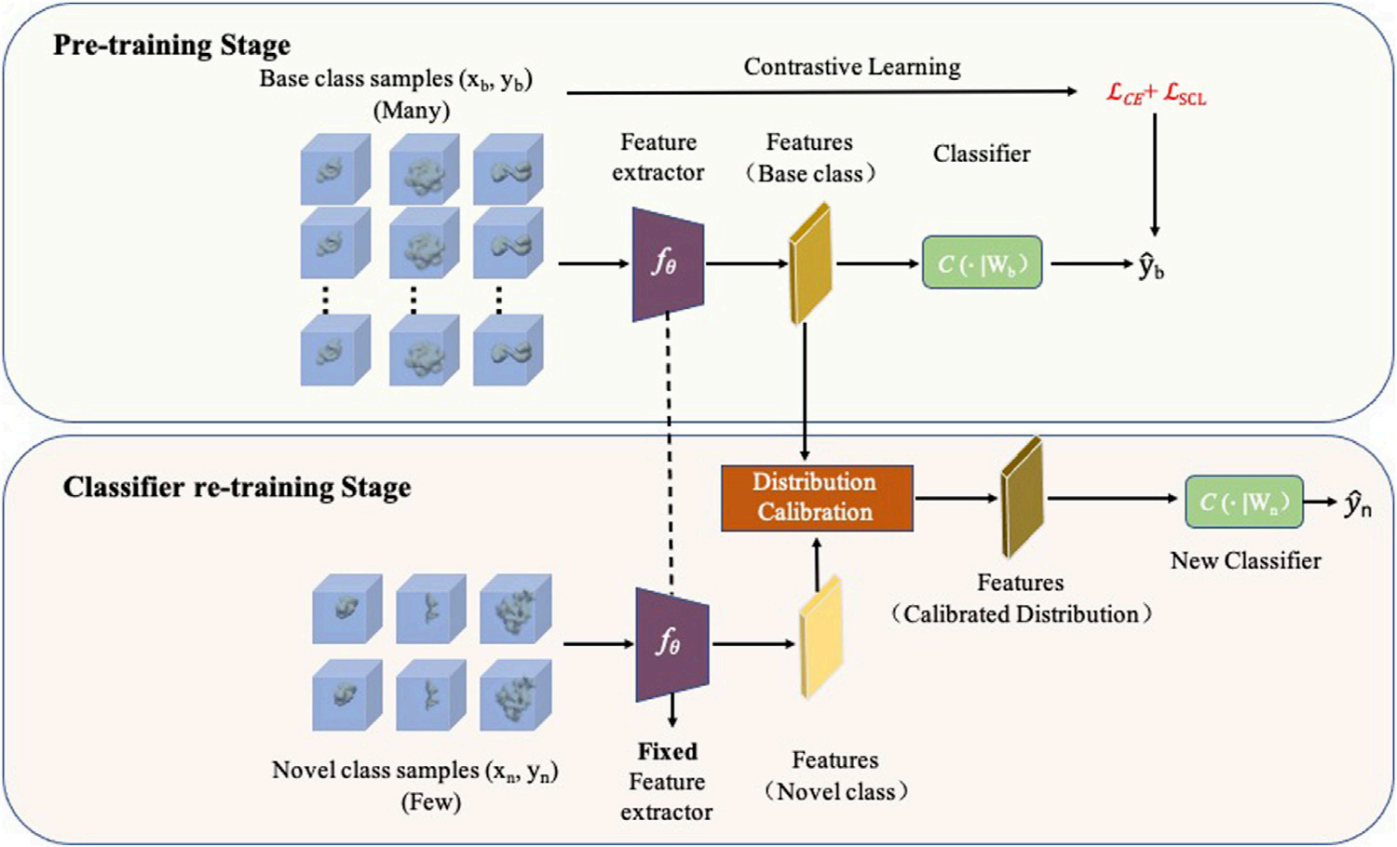
Framework of FSCC. FSCC follows a two-stage training strategy. The first training stage is a pre-training stage. FSCC pre-trains the model on a sufficient number of labeled macromolecules of base classes by contrastive learning. The second training stage is a classifier re-training stage. Here, FSCC re-trains the classifier with very few labeled macromolecules of novel class. To augment the inputs of the classifier, FSCC calibrates the distribution of novel classes and samples adequate features from calibrated distribution to re-train the classifier.

In this study, we propose a novel few-shot learning method for macromolecule classification of novel classes (named FSCC) (Figure 1). Combined with contrastive learning and distribution calibration, a two-stage training strategy is designed in FSCC to enhance the generalization ability of the model to novel macromolecules. First, FSCC uses contrastive learning (Khosla et al., 2020) to pre-train the model on a sufficient number of labeled macromolecules. This comes from the intuition that good generalization requires capturing the similarity between subtomograms in the same class and contrasting them with subtomograms in other classes. With contrastive learning, FSCC can pull together macromolecules belonging to the same class in the embedding space and separate macromolecules from different classes. Second, FSCC retrains the model to classify novel macromolecules. Specifically, FSCC freezes the parameters of the feature extractor of the pre-trained model. Then, based on distribution calibration (Yang et al., 2021b), FSCC retrains the classifier with a limited number of labeled macromolecules from novel classes. Distribution calibration is a kind of domain adaption method (Sun and Saenko, 2016). It can bridge the distribution gap between the source domain and target domain. Distribution calibration has been widely applied in high-level computer vision tasks such as object detection (Saito et al., 2019) and image retrieval (Su et al., 2019). In FSCC, the data distribution learned from a few macromolecules can be a biased distribution, which leads the model to become overfitted, whereas in the first pre-train stage, the data distribution learned from sufficient macromolecules is more accurate, which can alleviate such an overfitting problem. Thus, FSCC calibrates the distribution of the novel class by transferring the distribution statistics from the class with a sufficient number of macromolecules. After distribution calibration, FSCC samples an adequate number of features from the calibrated distribution to augment the inputs to the classifier.

To demonstrate the performance of FSCC, we tested FSCC on synthetic and real datasets. The results show that different from existing supervised deep learning–based macromolecule classification methods, FSCC has good generalization ability to novel macromolecules. FSCC can accurately classify novel macromolecules with very few labeled macromolecules. On synthetic datasets, FSCC achieves competitive performance when compared to the state-of-the-art (SOTA) method based on supervised deep learning. Specifically, on the synthetic dataset SHREC21, the F1-score of FSCC is 0.75, while the F1-score of SOTA is 0.73. To achieve such performance, FSCC uses only five labeled macromolecules per novel class. However, the SOTA method uses 1100 ∼ 1500 labeled macromolecules per novel class. On the real datasets, FSCC improves the accuracy by 5% ∼ 16% compared to the baseline model based on two-stage training. These demonstrate the good generalization ability of contrastive learning and calibration distribution to novel macromolecules.

## 2 Methods

### 2.1 Two-Stage Training Framework

#### 2.1.1 Problem Definition of Few-Shot Macromolecule Classification

Before introducing the framework of FSCC, we first briefly introduce the problem definition of the few-shot macromolecule classification. In the standard few-shot classification scenario (Li et al., 2021), there are two kinds of macromolecule datasets: a base dataset *D* ^base^ and a novel dataset *D* ^novel^. Let *C* ^base^ be the set of classes covered by *D* ^base^ and let *C* ^novel^ be the set of classes covered by *D* ^novel^, then we have *C* ^base^⋂*C* ^novel^ = ∅. The goal of the few-shot classification method is to use a model trained on *D* ^base^ to classify novel macromolecules of *D* ^novel^, given limited labeled macromolecules from *D* ^novel^. Here, in one few-shot classification task, the limited labeled macromolecules are defined as the support set and the unlabeled macromolecules, which are needed to be classified and are defined as the query set. The classes of the query set and support set are the same at the one few-shot task. According to the number of classes covered by the few-shot classification task and the number of labeled macromolecules per class in the support set, the few-shot task is named *N*-way-*K*-shot tasks. *N*-way-*K*-shot means there are *N* novel classes, and each class has *K* labeled macromolecules. Generally, N is set as 5 and K is set as 1 or 5.

#### 2.1.2 The Framework of FSCC

FSCC designs a two-stage training strategy: pre-training stage and classifier re-training stage to enhance the generalization ability of the model to novel macromolecules (Figure 1). In the pre-training stage, given a sufficient number of labeled macromolecules (*x*_*b*_, *y*_*b*_) from *D* ^base^, where *x*_*b*_ is a *I*_*n*×*n*×*n*_ 3D subtomogram image and *y*_*b*_ ∈ *C* ^base^, FSCC trains a feature extractor *f*_*θ*_ and a classifier *C*(⋅∣*W*_*b*_) to predict the class of macromolecules ${\hat{y}}_{b}$ . A good feature extractor *f*_*θ*_ should learn an embedding such that the features of macromolecules of the same class are close to each other, while the features of macromolecules of different classes are far apart. To learn such a feature extractor *f*_*θ*_, FSCC designs a weighted loss function L (Eq. (1)) based on contrastive learning (Jaiswal et al., 2020) (**Subsection 2.2**). This weighted loss function L contains two kinds of loss. The first one $L_{CE}$ (Eq. (2)) is a penalty of misclassified macromolecules. The other is class similarity–based loss $L_{\mathrm{SCL}}$ (Eq. (3)) which aims to find the similarities between the macromolecules of the same class and contrast them with macromolecules from other classes.

To enable the pre-trained model to classify novel macromolecules, given a few labeled macromolecules (*x*_*n*_, *y*_*n*_) from *D* ^novel^, FSCC freezes parameters of the feature extractor *f*_*θ*_ and retrains the classifier *C*(⋅∣*W*_*n*_). Training the classifier with very few labeled macromolecules is a challenge because the feature distribution learned from very few labeled macromolecules (*x*_*n*_, *y*_*n*_) can be a biased distribution (Yang et al., 2021a). This biased distribution cannot accurately reflect the ground-truth distribution of macromolecules of novel classes. Actually, the estimated distribution of the base dataset *D* ^base^ with a sufficient number of labeled macromolecules is more accurate than that of the novel dataset *D* ^novel^ with limited labeled macromolecules. Previous work has proved that semantically similar images have similar feature distributions (Burke, 2018). Thus, FSCC performs distribution calibration on extracted features of the input novel macromolecule *x*_*n*_ with learned knowledge from the base dataset *D* ^base^. The detailed description of distribution calibration is given in **Subsection 2.3**. After calibration distribution, FSCC samples adequate features from calibrated distribution to augment the input for the classifier.

### 2.2 Supervised Contrastive Learning

FSCC designs a weighted loss function L of the cross-entropy (CE) loss $L_{CE}$ and the supervised contrastive learning (SCL) loss $L_{\mathrm{SCL}}$ to pre-train the model. L is defined as

$$L=L_{CE}+\lambda L_{\mathrm{SCL}},$$ (1)

where *λ* is a weight coefficient of $L_{\mathrm{SCL}}$ . We suggest that *λ* is 0.05 based on the experimental results (**Subsection 3.3.2**).

**FIGURE 2:**
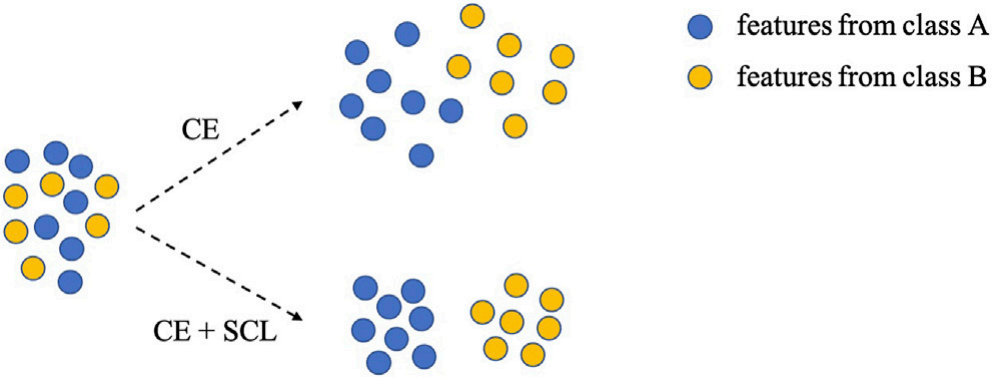
Conceptualization of cross-entropy loss $L_{CE}$ (eq. (2)) and FSCC loss L (eq. (1)). There are two kinds of extracted features of classes A and B. When adopting cross-entropy loss $L_{CE}$ to train the model, the inter-class distance is close. Instead, when adopting the loss of FSCC L , the inter-class distance increases, while intra-class distance reduces.

Cross-entropy loss $L_{CE}$ (Eq. (2)) is a commonly used loss function in existing supervised deep learning–based macromolecule classification methods. $L_{CE}$ is defined as

$$L_{CE}=-\frac{1}{M}\overset{M}{\underset{i=1}{\sum }}\overset{C_{b}}{\underset{c=1}{\sum }}y_{i,c}\cdot \log{\hat{y}}_{i,c},$$ (2)

where *M* is the number of input subtomograms in the mini-batch; *i* means the *i*-th macromolecule in this mini-batch; *c* means the class ID of base classes; *y*_*i*,*c*_ denotes the ground-truth label of input subtomograms, and ${\hat{y}}_{i,c}$ denotes the predicted class. Though CE loss $L_{CE}$ is good at learning the inter-class information, it only focuses on misclassified macromolecules, while ignoring the similarity between macromolecules from the same class. Therefore, the learned features of the same classes present scattered shape if the model is trained with only cross-entropy loss $L_{CE}$ (Figure 2).

Contrastive learning is one of the methods that have been widely used in self-supervised learning to enhance the generalization ability of the model recently (Jaiswal et al., 2020). The idea of contrastive learning is to find the similarities of samples of the same class by contrasting them with samples from other classes. In our task, with the label information, FSCC introduces supervised contrastive learning loss $L_{\mathrm{SCL}}$ to cluster macromolecules from the same class, while simultaneously pushing apart macromolecules from different classes. The supervised contrastive learning loss $L_{\mathrm{SCL}}$ is defined as

$$L_{\mathrm{SCL}}=\overset{N}{\underset{i=1}{\sum }}-\frac{1}{N_{y_{i}}-1}\overset{N}{\underset{j=1}{\sum }}I\left\{y_{i}=y_{j}\right\}\cdot \log\frac{\exp\left(f_{\theta }\left(x_{i}\right)\cdot f_{\theta }\left(x_{j}\right)/\tau \right)}{\sum_{k=1}^{N}I_{i\neq k}\exp\left(f_{\theta }\left(x_{i}\right)\cdot f_{\theta }\left(x_{k}\right)/\tau \right)},$$ (3)

where (*x*_*i*_, *y*_*i*_)(*i* = 1, 2, … , *N*) is the labeled macromolecules in one mini-batch; $N_{y_{i}}$ is the number of subtomograms that have the same label as *y*_*i*_; *f*_*θ*_ is the feature extractor of the model; and *τ* is a hyper-parameter that controls the separation of different classes. Referring the original contrastive learning loss, we set *τ* to 0.07 (Khosla et al., 2020). To ensure that the mini-batch macromolecules cover a different class of macromolecules, FSCC randomly samples a uniform number of macromolecules from each base class *C* ^base^. However, training the model with only supervised contrastive learning loss $L_{\mathrm{SCL}}$ can lead to slow convergence of the model. Therefore, in order to converge faster, FSCC designed a combined loss function of cross-entropy loss $L_{CE}$ and contrastive learning loss $L_{\mathrm{SCL}}$ (Figure 2).

### 2.3 Distribution Calibration

To apply the pre-trained model to classify novel macromolecules, we fix the parameters of the feature extractor *f*_*θ*_ and re-train the classifier *C*(⋅∣*W*_*n*_) with a limited number of labeled macromolecules of novel classes *C* ^novel^. The feature distribution learned from very few labeled macromolecules can be a biased distribution, which can make the model to be overfitted (Yang et al., 2021a). Actually, as the base dataset *D* ^base^ contains a sufficient number of labeled macromolecules, the learned feature distribution of base dataset *D* ^base^ is more accurate than that of the novel dataset *D* ^novel^. Previous works have proven that semantically similar images have similar distributions when the feature distribution follows a Gaussian distribution (Burke, 2018). Thus, to obtain a more accurate distribution of the novel dataset *D* ^novel^, FSCC calibrates the distribution of the novel dataset *D* ^novel^ by transferring the distribution statistics of the base dataset *D* ^base^ to the novel dataset *D* ^novel^.

The distribution calibration consists of four steps (Algorithm 1). In step 1, FSCC computes the mean and co-variance to describe the nearly-Gaussian feature distribution of each base class in *C* ^base^. The mean *μ* and co-variance Σ of each base class *i* are defined as follows:

$$\mu_{i}=\frac{\sum_{j=1}^{n_{i}}v_{j}}{n_{i}},\forall i\in C_{b},$$ (4)

$$\Sigma_{i}=\frac{1}{n_{i}-1}\overset{n_{i}}{\underset{j=1}{\sum }}\left(v_{j}-\mu_{i}\right){\left(\left(v_{j}-\mu_{i}\right)\right)}^{⊤},\forall i\in C_{b},$$ (5)

where v_*j*_ is a feature vector of the *j*-th macromolecules from the base class *i* and *n*_*i*_ is the total number of macromolecules in base class *i*.

In step 2, to make the feature distribution of the novel dataset *D* ^novel^ follow Gaussian distribution, FSCC transforms the feature vector $v={\left[v_{1},\ldots ,v_{m}\right]}^{⊤}$ of macromolecules of novel class *C* ^novel^ with the Tukey ladder of powers (Tukey, 1977). The computation is processed in each single dimension. The transformed feature vector $\tilde{v}={\left[{\tilde{v}}_{1},\ldots ,{\tilde{v}}_{m}\right]}^{⊤}$ is defined as

$$\tilde{v}=\left\{\begin{array}{cc} v^{\lambda },\; & \text{ if }\lambda \neq 0\\ \log\left(v\right)\; & \text{ if }\lambda =0 \end{array}\right.,$$ (6)

where *λ* is a hyperparameter that controls correcting the distribution. As suggested in the previous work, we set *λ* as 0.5 (Yang et al., 2021a).

In step 3, to search similar base classes to a novel class, FSCC computes the Euclidean metric between the transformed feature $\tilde{v}$ and the mean of base class *μ*_*i*_ of each base class *i* ∈ *C*_*b*_ (Eq. (7)). With a distance set $S_{\mathrm{distance}}$ , FSCC selects the k base classes that has a minimum distance to the transformed feature $\tilde{v}$ (Eq. (8)).

$$S_{\mathrm{distance}}=\left\{{\left\|\mu_{i}-\tilde{v}\right\|}^{2},\forall i\in C^{\text{base}}\right\},$$ (7)

$$S_{\mathrm{selected}}=\left\{i|{\left\|\mu_{i}-\tilde{v}\right\|}^{2}\in mink\left(S_{\mathrm{distance}}\right)\right\},$$ (8)

where *mink*(⋅) means selecting the k minimum distance from the distance set $S_{\mathrm{distance}}$ . $S_{\mathrm{selected}}$ covers the k nearest base classes to the transformed feature $\tilde{v}$ of the novel class.

In step 4, with the selected nearest k base classes $S_{\mathrm{selected}}$ , FSCC computes the statistics of the calibrated distribution of the novel class *C* ^novel^ (Eq. (9)–10). With the calibrated mean *μ*′ and co-variance Σ′ for each novel class, FSCC samples features from calibrated distribution to enrich the input for the classifier.

$$\mu^{\prime }=\frac{\sum_{i\in S_{n}}u_{i}+\tilde{v}}{k+1}.$$ (9)

$$\Sigma^{\prime }=\frac{\sum_{i\in S_{n}}\Sigma_{i}}{k}.$$ (10)

Algorithm 1 The training of the classifier in FSCC.

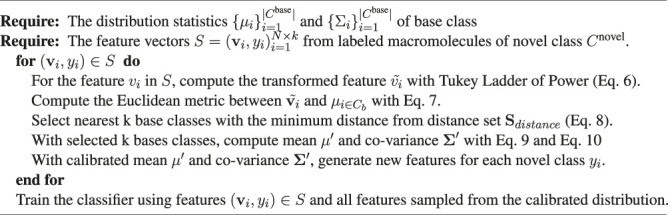

## 3 Experiments and Results

### 3.1 Data Preparation

**FIGURE 3:**
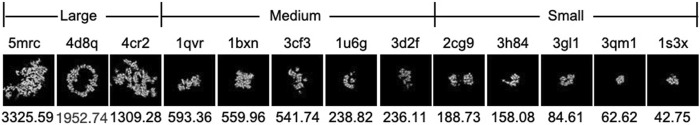
Density map and molecular weight (kDa) of each PDB ID in SHREC data. The top row is the PDB ID. The bottom row is the molecular weight (kDa). From left to right, the molecular weight of PDB decreases.

**FIGURE 4:**
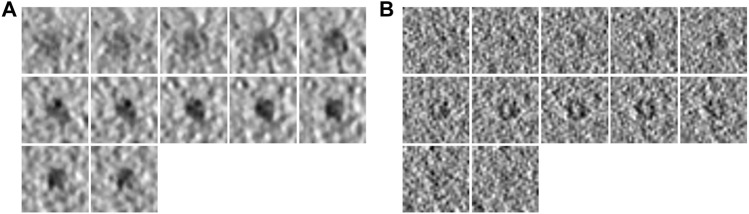
Example subtomograms of SHREC data. **(A)** Consecutive slices of a subtomogram of PDB ID 1bxn in SHREC19. **(B)** Consecutive slices of a subtomogram of PDB ID 1bxn in SHREC21.

To demonstrate the effectiveness of FSCC, it was tested on synthetic datasets and real datasets. We first introduce the synthetic datasets. There are two synthetic public datasets, which were released by SHREC in 2019 and 2021 (Gubins et al., 2019). For convenience, we named these two synthetic datasets SHREC19 and SHREC21. The raw SHREC dataset contains 10 reconstructed 3D tomograms and ground-truth information that record the localization and class of each macromolecule. The size of the 3D tomogram is 512 × 512 × 512 (1 voxel equals 1 nm). It contains thousands of macromolecules that are uniformly distributed. According to the molecular weight, macromolecules are grouped into large, medium, and small sizes by SHREC (Figure 3). We extract all subtomograms from 10 tomograms based on the ground-truth information. The extracted subtomogram is at the size of 32 × 32 × 32. Figure 4 shows an example of subtomograms of SHREC data. For SHREC19, there are 12 classes of macromolecules and 20785 macromolecules. The class distribution of macromolecules is uniform. Each class contains ∼1700 macromolecules. As published by SHREC, the SNR of SHREC19 is 0.02. For SHREC21, there are 13 classes of macromolecules and 16291 macromolecules. Each class contains ∼1300 macromolecules. In the N-way-K-shot classification tasks (N is set as 5), we randomly divided the SHREC data into the base dataset *D* ^base^ and novel dataset *D* ^novel^, with five classes of macromolecules. For SHREC19, there are seven classes of macromolecules as the base dataset *D* ^base^. For SHREC21, there are eight classes of macromolecules as the base dataset *D* ^base^. Both the base dataset *D* ^base^ and novel dataset *D* ^novel^ cover macromolecules of small, medium, and large sizes.

There are two real datasets in our work (Table 1). One real dataset covers seven classes of macromolecular structures published by Gao et al. (2020). For convenience, we name this dataset Dataset1. In Dataset1, there are 400 macromolecules, and each is reconstructed from the 2D tilt series with a size of 28 × 28 × 28. Another real dataset is generated by Guo et al. (2018). For convenience, we name this dataset Dataset2. Dataset2 covers five classes of macromolecular structures. Each class contains 200 macromolecules (28 × 28 × 28). As the real datasets contain a limited number of classes, the base dataset *D* ^base^ only contains a few class number of macromolecules if we set the number of novel classes (N) as 5 in the few-shot classification task. Thus, instead of setting N to 5 as in the synthetic dataset, we equally divide the classes of the real dataset into base class *D* ^base^ and novel class *D* ^novel^. For Dataset1, we randomly split the dataset into the base dataset *D* ^base^ with four classes of macromolecular structures and the novel dataset *D* ^novel^ with three classes of macromolecules. For Dataset2, we randomly split the dataset into the base dataset *D* ^base^ with three classes of macromolecular structures and the novel dataset *D* ^novel^ with two classes of macromolecules.

**TABLE 1:** Macromolecular structures covered in real datasets. The first line shows the macromolecular structures in Dataset1. The second line macromolecular structures in Dataset2.

Dataset Name	Base dataset				Novel dataset		
Dataset1	Rabbit muscle aldolase	Glutamate dehydrogenase	DNAB helicase-helicase	T20S proteasome	Apoferritin	Hemagglutinin	Insulin-bound insulin receptor
Dataset2	Mitochondrial membrane	Ribosome	26S proteasome		Double capped proteasome	TRiC	

### 3.2 Implementation Details

**FIGURE 5:**
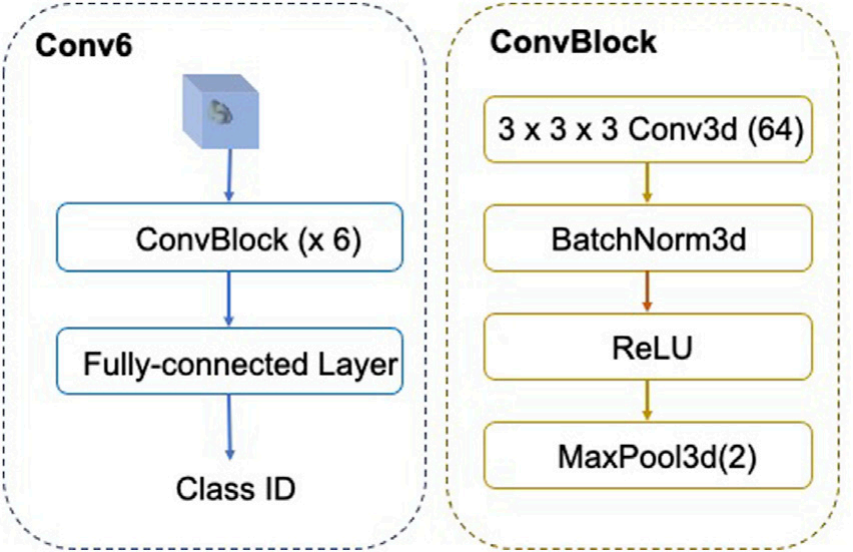
Architecture of FSCC Conv-6. In Conv-6, the feature extractor *f*_*θ*_ comprises six ConvBlocks and the classifier is the fully connected layer.

The architecture of FSCC is named as Conv-6 (Figure 5). The input of Conv-6 is a 3d subtomogram *x*_*i*_, and the output is the class ID $\hat{y}$ of macromolecules. In Conv-6, the feature extractor *f*_*θ*_ comprises six ConvBlocks, and the classifier is the fully connected layer. Each ConvBlock is a composition of a 3d convolution layer, a batch normalization layer, a ReLu, and a 3d pooling layer. FSCC is implemented with Pytorch and trained on the GTX 2080ti GPU. In the pre-training stage, we train the Conv-6 with all macromolecules from the base dataset *D* ^base^. The optimizer is Adam; the initial value of the learning rate is 0.05. When training with the synthetic datasets, the batch size is 128 and the training epoch is 5. When training with the real datasets, due to the small number of labeled macromolecules, to make the model converge, we set the batch size as 32 and the training epoch as 50. In the fine-tuning stage, we fixed the parameters of the feature extractor of Conv6 *f*_*θ*_ and re-trained the classifier *C*(⋅∣*W*_*n*_) with very few limited macromolecules of novel class *C* ^novel^. In each few-shot classification task, we have *N* × *K* limited labeled macromolecules (support set) to train the classifier and *N* × *Q* unlabeled macromolecules (query set) to predict. N is equal to the number of novel class *C* ^novel^. K is generally set to 1 or 5. For the synthetic datasets, N is 5. For the real dataset Dataset1, N is 3 and Dataset2 N is 2. All macromolecules in the support set and query set are randomly sampled from the novel dataset *D* ^novel^. The classes of the query set and support set are the same at one few-shot classification task. To demonstrate the stability of FSCC, we tested it on randomly sampled 100 few-shot classification tasks. The performance of FSCC reflects in the mean classification accuracy (Eq. (12)) and F1 score (Eq. (11)) for these 100 tasks. In Eq. (12) and Eq. (11)), TP means true positive, TN means true negative, FN means false negative, and FP means false positive.

$$accuracy=\frac{\text{TP}+\text{TN}}{\text{TP}+\text{FN}+\text{FP}+\text{TN}}.$$ (11)

$$F1=\frac{2\text{TP}}{2\text{TP}+\text{FN}+\text{FP}}.$$ (12)

### 3.3 Results on Synthetic Data

#### 3.3.1 The Classification Results on Synthetic Datasets

Here, we show the classification performance of FSCC on the synthetic datasets (Table 2). We tested FSCC and two popular fine-tuning–based methods on the few-shot classification task with SHREC19 and SHREC21. These two methods are named Baseline (Chen et al., 2019) and Baseline++ (Chen et al., 2019) in the original. Baseline and Baseline++ use the same architecture network (Figure 5) as the FSCC and adopt a two-stage training strategy. Baseline is a standard fine-tuning method. Baseline++ is the same as the original Baseline, except for the training of the classifier. Baseline++ trains the classifier base on cosine distance similarity to explicitly reduce intra-class variations. The pre-training strategy of Baseline and Baseline++ is the same as that of FSCC. In the fine-tuning stage, Baseline and Baseline++ re-train the classifier with 10 epochs. In each epoch, Baseline and Baseline++ randomly sample *N* × *K* labeled macromolecules to re-train the classifier. Thus, Baseline and Baseline++ methods use 10 × *K* labeled macromolecules of novel macromolecules per class. Concretely, Baseline and Baseline++ use 50 labeled macromolecules per class when adapting the model to 5-way-5-shot classification tasks of novel macromolecules and 10 labeled macromolecules per class when adapting the model to 5-way-1-shot tasks of novel macromolecules. In contrast to Baseline and Baseline++ methods, FSCC can re-train the classifier in one few-shot classification task. This means FSCC only needs five labeled macromolecules on 5-way-5-shot classification tasks of novel macromolecules and one labeled sample on the 5-way-1-shot classification tasks. In Table 2, we report the classification performance and the number of labeled macromolecules of each novel class that are needed in the fine-tuning stage. The classification performance is demonstrated by the mean and variance of classification accuracy (Eq. (12)) on 100 few-shot classification tasks. The number of labeled macromolecules is shown in parentheses after the classification accuracy. Due to the 5-way-5-shot task providing more labeled macromolecules than that of the 5-way-1-shot task, the classification performance of FSCC on the 5-way-5-shot task is higher than that of the 5-way-1-shot task. Compared with Baseline and Baseline++ methods, on SHREC19, our method improves the accuracy by 3.86% when there are five labeled macromolecules per class and 5.44% when there is one labeled sample per class. On SHREC21, our method improves the accuracy by 4.71% when there are five labeled macromolecules per class and 9.09% when there is one labeled sample per class.

**TABLE 2:** Classification performance of FSCC and SOTA methods on the synthetic dataset. The classification performance is measured by classification accuracy, followed by the number of labeled training macromolecules in parentheses.

Methods	SHREC19		SHREC21	
	5-Way-5-Shot	5-Way-1-Shot	5-Way-5-Shot	5-Way-1-Shot
Baseline (Chen et al., 2019)	73.14 ± 0.47%(50)	65.12 ± 0.74%(10)	71.36 ± 1.19%(50)	59.15 ± 1.70%(10)
Baseline++ (Chen et al., 2019)	75.32 ± 0.37%(50)	66.65 ± 0.67%(10)	73.64 ± 1.04%(50)	65.19 ± 1.02%(10)
FSCC (ours)	77.03 ± 1.21% (5)	70.56 ± 1.61% (1)	76.07 ± 1.03% (5)	68.24 ± 1.03% (1)

We also compared FSCC with the state-of-the-art (SOTA) performance of supervised deep learning–based methods on SHREC19 and SHREC21. Table 3 shows the F1-score (Eq. (11)) and the number of labeled training macromolecules for each class of macromolecules of FSCC and SOTA. From the average F1 score, we can see that FSCC can achieve the classification performance of SOTA methods with five labeled macromolecules per novel class. However, the SOTA method uses a significantly larger number of 1100 ∼ 1500 labeled macromolecules per novel class. It is worth emphasizing that the SOTA results are published by the SHREC contest. SHREC publics the F1-score of each macromolecule of many popular supervised deep learning–based methods. Here, the SHREC–SOTA means the highest F1 score of each class of macromolecule. Thus, SHREC–SOTA comes from different supervised deep learning–based methods. For most methods, there has been no access to source code or pretrained models. Table 3 only reports the performance of macromolecular structures from novel class *C* ^novel^. In SHREC19 and SHREC21, the PDB ID of macromolecules in the novel class is 1u6g, 3cf3, 3gl1, 3qm1, 4d8q, and 4v49. 4d8q is only covered in SHREC19, and 4v49 is only covered in SHREC21. According to the molecular weight, 3g1 and 3qm1 are macromolecules of small size, 1u6g and 3cf3 are macromolecules of medium size, and 4d8q and 4v4g are macromolecules of large size. The result shows that for both methods, macromolecules of large size are easy to be classified. When the size decreases, the classification accuracy also decreases. The SHREC contest contains localization and classification tasks. In SHREC19, SOTA methods first localized the macromolecules and then classified them. Thus, the macromolecules may not be in the center of the input subtomograms. This makes sense that FSCC has higher classification accuracy because the input subtomograms are extracted according to the ground-truth localization. In SHREC21, SOTA methods adopt the end-to-end pixel classification–based model to obtain the class of macromolecular structures. For macromolecules of large size, the classification accuracy of FSCC is close to SOTA methods. Even for macromolecules of small size such as 3qm1, FSCC improves the classification performance by 0.31. This is because FSCC classifies novel macromolecules according to the statistic of macromolecules of the base class. In the base dataset, 1s3x is similar to 3qm1. Thus, the feature vectors of 1s3x are similar to those of 3qm1, which leads FSCC to classify 3qm1 with higher accuracy than SOTA.

**TABLE 3:** Classification performance of FSCC and SOTA methods on the synthetic dataset. The classification performance is measured by the F1 score, followed by the number of labeled training macromolecules in parentheses.

PDB ID	SHREC19						SHREC21					
	1u6g	3cf3	3gl1	3qm1	4d8q	avg. F1	1u6g	3cf3	3gl1	3qm1	4v49	avg. F1
SHREC-SOTA	0.52 (1556)	0.78 (1689)	0.31 (1539)	0.19 (1509)	0.95 (1556)	0.55	0.73 (1268)	0.96 (1182)	0.51 (1200)	0.48 (1148)	0.97 (1376)	0.73
5-way-5-shot (FSCC)	0.70 (5)	0.93 (5)	0.47 (5)	0.69 (5)	0.99 (5)	0.75	0.65 (5)	0.97 (5)	0.38 (5)	0.79 (5)	0.97 (5)	0.75
5-way-1-shot (FSCC)	0.59 (1)	0.88 (1)	0.39 (1)	0.66 (1)	0.99 (1)	0.70	0.48 (1)	0.89 (1)	0.37 (1)	0.65 (1)	0.93 (1)	0.66

#### 3.3.2 Ablation Studies

FSCC contains two key components: contrastive learning (**Subsection 2.2**) and distribution calibration (**Subsection 2.3**). Here, we performed an ablation study on SHREC19 to explore the contribution of each key component. In Table 4, there are four CNN models tested in the ablation study. First, we tested the base CNN model (Baseline) without contrastive learning and distribution calibration. Second, we added distribution calibration to the Baseline to test the contribution of distributed calibration. Third, we added contrastive learning to the Baseline to test the contribution of contrastive learning. Compared to Baseline, distribution calibration improves by 5.65% on 5-way-1-shot classification tasks. The aim of the distribution calibration is to calibrate the biased distribution learned from very few labeled macromolecules of novel class. Therefore, in the case of fewer labeled macromolecules, it is reasonable that the distribution calibration can more significantly improve the accuracy of image classification. The last model is our FSCC model. These results show the contribution of contrastive learning and distribution calibration to the few-shot macromolecule classification.

**TABLE 4:** Ablation study on 5-way-5-shot and 5-way-1-shot classification tasks with SHREC19.

Calibrated distribution	Contrastive learning	5-Way-5-Shot	5-Way-1-Shot
No	No	73.14 ± 0.47%	65.12 ± 0.74%
Yes	No	75.97 ± 0.89%	70.77 ± 0.19%
No	Yes	74.32 ± 0.12%	68.44 ± 0.14%
Yes	Yes	77.12 ± 0.21%	70.59 ± 0.61%

**FIGURE 6:**
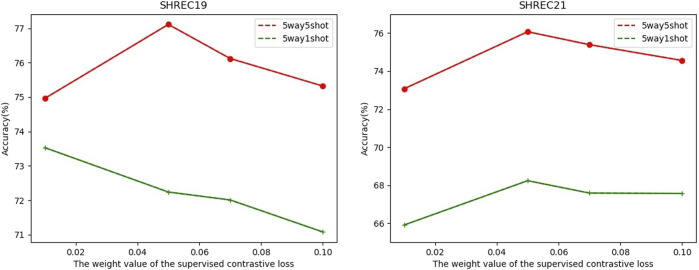
Relationship between classification accuracy with the weight value *λ* of the supervised contrastive loss $L_{\mathrm{SCL}}$ .

In FSCC, we adopted cross-entropy loss and contrastive learning loss in the pre-training stage. The total loss function is shown in Eq. (1). Here, *λ* is a hyperparameter to control the component of supervised contrastive learning loss $L_{\mathrm{SCL}}$ . On the SHREC19 and SHREC21 datasets, we test the relationship between the classification performance and the value of *λ* (Figure 6). To ensure that the cross-entropy loss and the supervised contrastive loss are of the same order of magnitude, the *λ* is set to 0.01, 0.05, 0.07, and 0.1. The results show that except for 5-way-1-shot on SHREC19, *λ* = 0.05 has the best test accuracy across all experimental settings. The test accuracy for the 5-way-1-shot task on SHREC19 differs by 1.29% when *λ* equals 0.01 and 0.05. According to all hyperparameter experiments of *λ*, we recommend that the *λ* can be set as 0.05.

### 3.4 Results on Real Data

Here, we tested FSCC on two real datasets. Table 5 shows the classification performance and training macromolecules of FSCC and baseline models (Baseline and Baseline++) on Dataset1 and Dataset2. The classification performance is represented by the mean and variance of the classification accuracy of randomly constructed 100 few-shot classification tasks. As the real datasets only cover a few classes of macromolecular structures and hundreds of macromolecules per class, the stability of the feature extractor of all pre-trained models is poor. This results in a variance of classification accuracy greater than 1%. In addition, the insufficient labeled macromolecules of the base class lead to poor generalization ability of Baseline and Baseline++. Thus, the classification performance of Baseline and Baseline++ is poor to novel macromolecules. Compared with Baseline methods, for Dataset1, FSCC significantly improves classification accuracy by 16.84% on 3-way-1-shot classification tasks and 5.96% on 3-way-5-shot classification tasks. For Dataset2, FSCC significantly improves classification accuracy by 13.1% on 2-way-1-shot classification tasks and 7.76% on 2-way-5-shot classification tasks.

**TABLE 5:** Classification performance of FSCC on real datasets. The classification performance is measured by classification accuracy, followed by the number of labeled training macromolecules in parentheses.

	Dataset1		Dataset2	
	3-Way-5-Shot	3-Way-1-Shot	2-Way-5-Shot	2-Way-1-Shot
Baseline	69.96 ± 1.41%(50)	64.44 ± 1.91%(10)	67.60 ± 0.94%(50)	65.27 ± 0.95%(10)
Baseline++	69.22 ± 1.33%(50)	65.44 ± 0.85%(10)	68.17 ± 1.06%(50)	66.93 ± 1.74%(10)
FSCC	86.80 ± 1.27% (5)	70.40 ± 1.82% (1)	80.70 ± 1.28% (5)	73.03 ± 1.49% (1)

## 4 Discussion and Conclusion

The classification of subtomograms is a key step to recover macromolecular structures captured by cryo-ET. Although supervised deep learning–based methods have improved the classification accuracy, they have limited ability to classify novel macromolecules. To adapt the model to a novel class of macromolecules, the trained model needed to be re-trained with massive labeled macromolecules of the novel class. However, it is inefficient and undesirable in practice as labeling the sample is time-consuming and laborious. In this work, we proposed a few-shot learning-based macromolecule classification method named FSCC. Different from the existing supervised deep learning–based methods, FSCC can classify novel macromolecules with very few labeled macromolecules. Based on a two-step training strategy, FSCC first pre-trained the model with supervised contrastive learning on the base dataset with a sufficient number of labeled macromolecules. Supervised contrastive learning can help enhance the generalization ability and stability of the model. Then, FSCC re-trains the classifier with distribution calibration to enable the model to classify novel macromolecules. The results on synthetic datasets demonstrate that compared to SOTA of supervised deep learning–based methods, FSCC can achieve competitive performance given only five labeled macromolecules per novel class. However, the SOTA method needs 1100 ∼ 1500 labeled training macromolecules per novel class. On the synthetic dataset SHREC19 (SNR = 0.02), compared to the popular fine-tuning–based few-shot classification method, FSCC improves classification accuracy by 3.89% on 5-way-5-shot tasks and by 5.44% on 5-way-1-shot tasks. On real datasets, compared to popular fine-tuning–based few-shot classification methods, FSCC improves classification accuracy by 5% ∼ 7% when there are five labeled macromolecules per class of novel macromolecules. FSCC significantly improves classification accuracy by 13% ∼ 16% when there is only one labeled sample per class of novel macromolecules.

## Data Availability

The original contributions presented in the study are included in the article/Supplementary Material; further inquiries can be directed to the corresponding authors.
